# Wood Flour Modified by Poly(furfuryl alcohol) as a Filler in Rigid Polyurethane Foams: Effect on Water Uptake

**DOI:** 10.3390/polym14245510

**Published:** 2022-12-16

**Authors:** Andrey Acosta, Arthur B. Aramburu, Rafael Beltrame, Darci A. Gatto, Sandro Amico, Jalel Labidi, Rafael de Avila Delucis

**Affiliations:** 1 Post-Graduate Program in Mining, Metallurgical and Materials Engineering, Federal University of Rio Grande do Sul, Porto Alegre 91509-900, Brazil; 2 Post-Graduate Program in Materials Science and Engineering, Federal University of Pelotas, Pelotas 96010-610, Brazil; 3 Post-Graduate Program in Environmental Sciences, Federal University of Pelotas, Pelotas 96010-610, Brazil; 4 Environmental and Chemical Engineering Department, University of the Basque Country, Plaza de Europa 1, 20018 San Sebastián, Spain

**Keywords:** polyurethane foam, furfuryl alcohol, water uptake, wood flour

## Abstract

The use of lignocellulosic fillers in rigid polyurethane foams (RPUFs) has been receiving great attention due to their good mechanical and insulation properties and the high sustainable appeal of the obtained cellular polymers, although high water uptakes are found in most of these systems. To mitigate this detrimental effect, RPUFs filled with wood flour (2.5% wt) were fabricated with the addition of furfuryl alcohol (FA) to create a polymer grafted with the wood filler. Two concentrations of FA (10 wt% and 15 wt%) were investigated in relation to the wood flour, and the RPUFs were characterized for cell morphology, density, compressive properties, thermal stability, and water uptake. The introduction of wood flour as a filler decreased the cell size and increased the anisotropy index of the RPUFs and, in addition to that, the FA grafting increased these effects even more. In general, there were no significant changes in both mechanical and thermal properties ascribed to the incorporation of the fillers. On the other hand, a reduction of up to 200% in water uptake was ascribed to the FA-treated fillers.

## 1. Introduction

Rigid polyurethane foams (RPUFs) are three-dimensional, highly cross-linked porous materials with low density, high dimensional stability, and low thermal conductivity [1]. These desirable properties make RPUFs a versatile class of polymer materials with a large range of applications, including insulation and manufacture of furniture and sporting goods [2]. However, RPUFs are prepared through the reaction between an isocyanate and a polyol, which are often derived from petroleum-based resources [3]. In this sense, the use of renewable resources in RPUFs as substitutes for petroleum-based compounds has been receiving great attention, especially due to the use of bio-based oils as polyols, distilled water as blowing agents, and chemical additives based on glycerol, among others [4,5,6].

Furthermore, the use of natural fillers also plays an important role in this field due to their positive influence on several properties of RPUFs. In this sense, forest and agricultural residues stand out due to their high availability, low price, and high renewability [7], as well as good thermal [8] and acoustic [9] insulating properties. Furthermore, free hydroxyl groups (–OH) on the surface of these lignocellulosic matrixes give a high host chemical compatibility with RPUFs, since they are prone for binding to isocyanate groups (–NCO) from the polymer matrix [10].

Among the most studied bio-based residues, wood flour is one of the most widely used fillers as reinforcement for polymer composites. For instance, Chanlert and Ruamcharoen [9] achieved improved sound absorption properties in RPUFs using a rubber wood-based sawdust as a filler. In a more recent study, De Luca Bossa and co-workers [7] reported increases in thermal stability and some mechanical properties, which were attributed to the incorporation of vegetable fillers, namely powdered cellulose and walnut shells. In the same way, Delucis and co-workers [11,12,13] reported the use of wood, wood bark, pine cones, pine needles, powdered kraft lignin, and paper sludge as fillers in castor oil-based RPUFs. These authors reported increases in thermal and mechanical properties, accompanied by decreases in cell sizes. On the other hand, these same authors reported losses in hygroscopic properties.

The high water uptake of RPUFs reinforced with natural fillers is mostly related to both the disruption of cell edges and the hydrophilic nature of the inserted fillers [11]. During the RPUF rising, the presence of moist air in cellular pores can also increase the cure rate through the reaction of NCO groups with water, which favors the growth of the polymer network through polyurea formation and generation of encapsulated CO_2_ [14]. However, the absorption of moisture in cured RPUFs may bring detrimental effects to the whole cellular polymer since the NCO groups may react with water molecules in the air at room temperature, releasing CO_2_ and reducing the RPUF performance [15]. The contact of water with RPUF systems may also cause the hydrolysis of ester groups derived from the polyol [16]. Therefore, low water uptake is linked to high durability and high thermal conductivity in RPUFs. Thus, increasing the hygroscopic performance of a RPUF is a way for minimizing its long-term performance variation. In addition, moisture absorbed by the filler from a RPUF may bring a detrimental effect on its thermal conductivity since liquid water has 10 times higher thermal conductivity than a conventional RPUF [11].

In conventional PU-based composite materials, surface treatments of lignocellulosic materials are widely applied in order to mitigate their hydrophilic character since these processes may enhance interfacial adhesion of natural fillers to the polymer matrix, leading to increases in physical performances [17,18]. In this sense, many authors reported solutions to increase the compatibility of lignocellulosic fillers and PU systems. Kılınç and co-workers [17] used alkaline and silane modifications to improve the compatibility of wood flour and a PU elastomeric composite. Członka and co-workers [2] applied a silanization process in walnut shells for reaching improvements in the mechanical and thermal properties of their filled RPUFs. Finally, Bradai and co-workers [19] applied acetylation as a pre-treatment on different wood-based resources, namely milled wood sieved at different particle sizes (<0.106 mm, 0.1–0.3 mm and 0.3–0.5 mm), kraft chemical pulp, and microcrystalline cellulose. These materials were then inserted into the RPUFs, yielding different improvements in mechanical and thermal properties.

In this sense, furfural is a platform chemical (i.e., this is a feedstock for producing various high value-added products), being one of the main derivatives from hemicelluloses and a natural precursor to furan-based chemicals, such as furfuryl alcohol [20,21,22]. furfuryl alcohol (FA) grafting is a widely used process for wood treatment (wood furfurylation), that consists of impregnating FA into the wood structure and inducing an in situ polymerization into poly(furfuryl alcohol). In this study, wood flour was treated at different FA concentrations and then added to RPUF systems to improve their hygroscopic performance.

## 2. Materials and Methods

### 2.1. Fillers Preparation and Furfurylation

Wood flake leftovers from the processing of pinewood logs were collected in a sawmill in Southern Brazil. This forest-based resource was oven dried at 50 °C until reaching a constant weight, milled using a Wiley mill, and then sieved using a 100-mesh screen. The resulting wood flour was characterized via wet-chemical analysis to obtain ethanol-toluene extractives [23], acid-insoluble lignin [24], ashes [25], and holocellulose (remaining mass percentage) contents.

The furfurylation process occurred by the addition of a high-purity (98 wt%) furfuryl alcohol (FA) acquired from Sigma Aldrich at two different concentrations (c.a. 10 and 15 wt% in relation to the wood flour weight). A high-purity (98 wt%) maleic anhydride (MA) acquired from Sigma Aldrich was added as a catalyst at a concentration of 1.5 wt% in relation to the FA weight. The FA, MA, and wood flour fractions were mixed with 500 mL of water and then magnetically homogenized for 15 min. Afterward, the treated wood flour was oven heated at 60 °C for 24 h. Apparent contact angle measurements were performed using a CREVIS goniometer. For that, a 5-mL deionized water droplet was placed on uniform pellets (diameter: 10 mm) manufactured by a compressive load of 80 kN applied for 15 s.

### 2.2. Preparation of the RPUFs

Castor oil and glycerin were used at a 3:1 weight ratio as a bio-based polyol. Isotane DM, which is a polymer methylene diphenyl diisocyanate (p-MDI), was used as an NCO source. Poly-ethylene glycol (PEG-400), silicon oil, and dimethylbenzylamine were used as a chain extender, a surfactant, and a catalyst, respectively.

The RPUF parts were produced by the single-shot method using two components (A and B), keeping a constant NCO/OH ratio of 1.2. Component A (polyol mixture) consisted of a simple mixture of castor oil (67.5 parts/g), distilled water (4 parts/g), glycerol (22.5 parts/g), PEG-400 (10 parts/g), silicon oil (2.5 parts/g), and filler, which were stirred together at 1000 rpm for 60 s and then with component B (p-MDI and amine (1 part/g)) for an extra 60 s. All RPUFs freely rose inside opened wooden boxes and were let to cure at 60 °C for 2 h and post-cure for two weeks at room temperature. Table 1 shows the adopted nomenclature of the studied RPUFs.

### 2.3. Characterization of the RPUFs

All fabricated RPUFs were milled and analyzed using a Fourier-transform infrared (FTIR) spectroscopy coupled with an attenuated total reflection device (ATR) in an IRSpirit equipment (Shimadzu^®^ brand). The reported spectrum of each sample is the average of 32 scans within the 600–4000 cm^−1^ range at a scan interval of 4 cm^−1^. The FTIR data were smoothed by applying a Savitzky–Golay filter (second degree polynomial at an interval of 12). Thermal stability was evaluated using a thermogravimetric analyzer (TG) at a heating rate of 10 °C.min^−1^ from room temperature (c.a. 20 °C) to 800 °C, which was carried out using a TGA-1000 Navas equipment under a nitrogen atmosphere. Surface morphology was analyzed perpendicular to the rise direction using a scanning electron microscopy (SEM) in a MA10 equipment (Zeiss Evo brand) operating at 3 kV. Average cell size and anisotropy index were measured using the ImageJ software, as described by Delucis and his co-workers [11].

Seven samples (5.0 × 5.0 × 2.5 cm^3^) of each group were taken to determine apparent density (ASTM D1622) and mechanical properties under compression parallel to the rise direction (ASTM D 1621). The compressive tests were carried out at a 2.5 mm.min^−1^ speed using a 23-5D Emic universal testing machine. Finally, water uptake measurements were performed in 10 samples per group, which had the dimensions of 5.0 × 5.0 × 2.5 cm^3^ (smaller dimension oriented in the rise direction). The specimens were totally immersed in distilled water at room temperature (around 20 °C) in accordance with the ASTM D570. Then, the mass gain was monitored every 30 min until reaching a total soaking time of 300 min.

### 2.4. Statistical Analyses

All data, except the chemical and water uptake results, were subjected to homogeneity of variance and data normality tests. Later, ANOVA tests were carried out and, whenever the null hypothesis was rejected, Tukey tests were used to compare the means. All statistical analyses were implemented at a significance level of 5%.

## 3. Results and Discussion

### 3.1. Filler’s Properties

**Figure 1:**
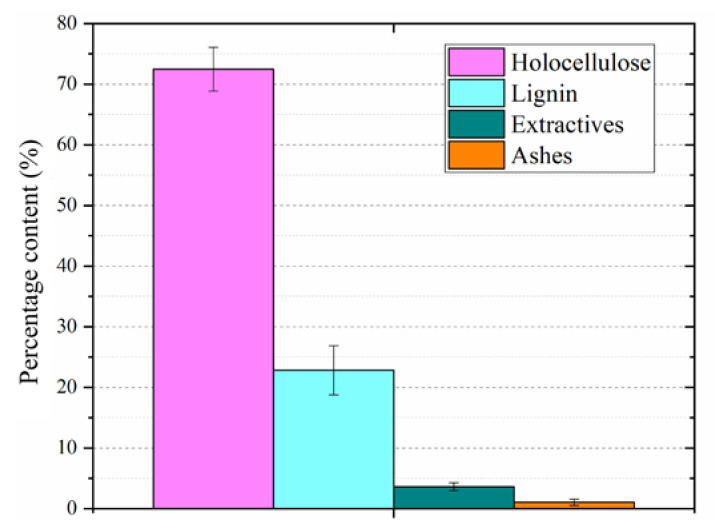
Chemical constitution of the wood flour.

The high holocellulose content (above 70 wt%) obtained for the studied wood flour (shown in Figure 1) is comparable to other holocellulose-rich biomasses, such as corncob and cork [26]. This high holocellulose content may impact several conversion processes applied to wood products due to the high number of strong intra-macromolecular covalent bonds between sugar units from cellulose and hemicelluloses, requiring a high energy amount to reach homolytic breaks [11]. In addition, the high holocellulose content may be of interest due to free OH groups from the amorphous polysaccharides, such has amorphous cellulose and hemicelluloses, which are prone to form urethane linkages with NCO groups from the p-MDI.

**Figure 2:**
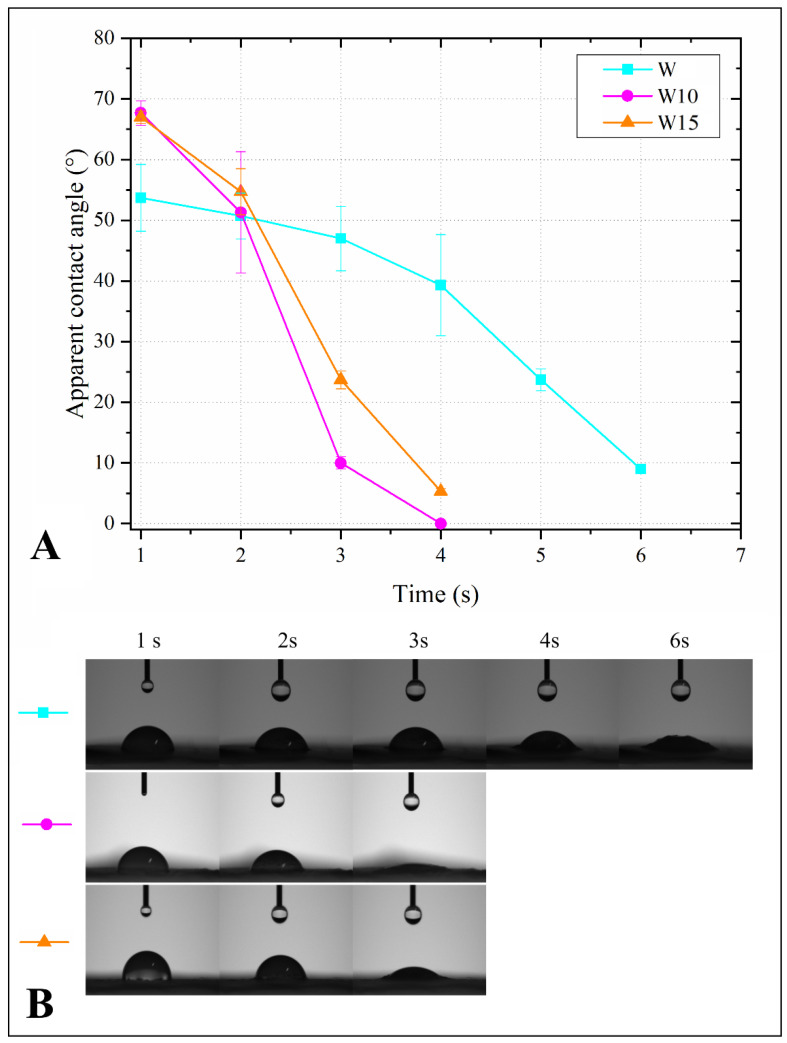
Apparent contact angle evolution in terms of mean values (**A**) and images (**B**) for the studied fillers.

The contact angle kinetics observed for each filler are shown in Figure 2. Both furfurylated woods presented similar profiles of drop absorption, in which a sharp decrease occurred along the first 4 s. Compared to the pristine wood flour, the FA-treated ones presented more unstable profiles, which indicates that they presented a smaller surface hydrophobicity. The chemical affinity of milled lignocellulosic products with water is mostly attributed to highly polar chemical groups, such as hydroxyls and methoxyls, which may interact with water via hydrogen bonds. Therefore, the changes in surface hydrophobicity imparted by the treatments are probably associated with modifications in polar chemical groups presented in cellulose, lignin, and hemicelluloses from the wood caused by the FA polymerization. Although some recent studies have proposed some explanations for the in situ polymerization of FA inside wood [27], this mechanism has not been fully clarified yet.

**Figure 3:**
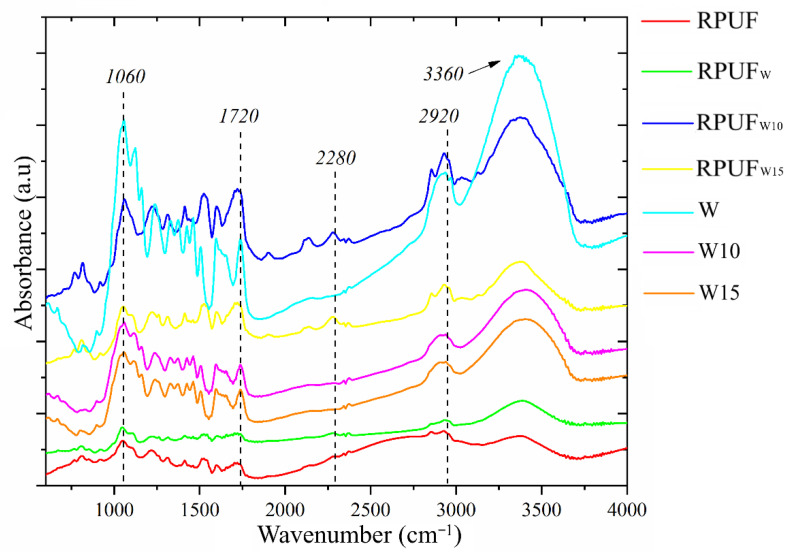
FTIR spectra of the studied fillers and RPUFs.

Figure 3 shows the infrared spectra of the treated and untreated wood flours and their respective RPUFs. The peaks at 1060 cm^−1^, 2900 cm^−1^, and 3360 cm^−1^, which are associated with the stretching vibration of C-O-C [28], CH_3_ [29], and N-H [29] groups, respectively, are found for all the studied RPUFs and indicate a good polymer formation in all cases. The FA treatment is evidenced by the attenuation in the peak at 1700 cm^−1^ [30], which is found for both FA-treated wood flours (RPUF_W10_ and RPUF_W15_). Furthermore, the prominent peak at 2280 cm^−1^, found in both RPUF_W10_ and RPUF_W15_, are related to unreacted NCO groups. This indicates that the FA blocks some OH groups from the wood flour, preventing the formation of urethane linkages with NCO groups from the p-MDI.

### 3.2. Foam Morphology and Density

**Figure 4:**
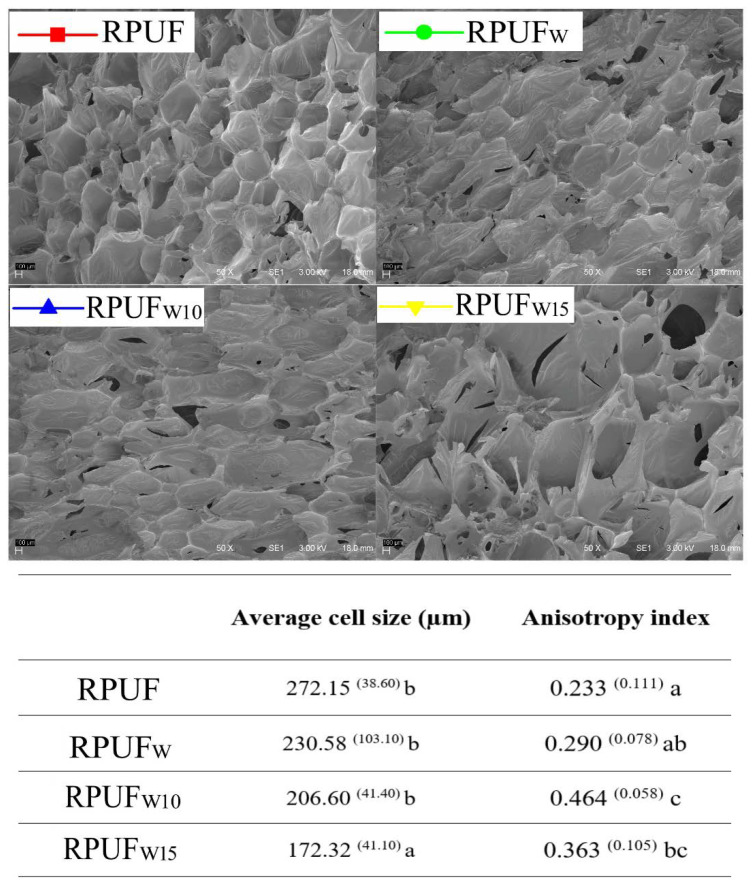
SEM images and morphological properties of the studied RPUFs. Different letters represent statistically different means. Different letters (e.g., a, b and c) represent statistically different means and equal letters represent no statistically different means (e.g., a and ab).

The SEM images shown in Figure 4 indicate that all fillers are well dispersed into the RPUF cell structure since no agglomerates are found. In addition, there are no noticeable signal of cell disruptions associated with the filler insertion, which indicates a good filler/matrix interaction. Only the filler treated with 15% of FA induces a significant increase in both RPUF cell length and anisotropy index. The elongation of polymer cells is associated with a vigorous cell growth, which is normally caused by chemically compatible fillers [31]. This indicates that the treatment with 15% of FA probably yields a filler with a higher host compatibility in the RPUF system when compared to the other fillers.

**Figure 5:**
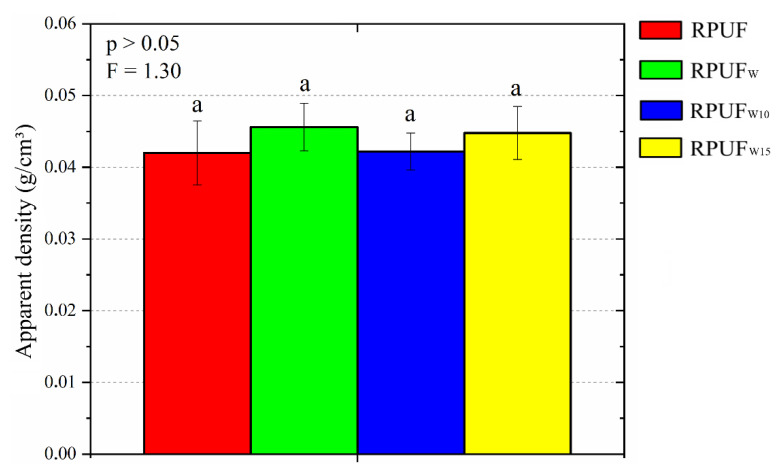
Apparent density of the studied RPUFs. RPUF is pure rigid polyurethane foam, RPUFw is wood flour reinforced foam, RPUF_W10_ is wood flour reinforced foam treated with 10% furfuryl alcohol, and RPUF_W15_ is wood flour reinforced foam treated with 15% furfuryl alcohol. Different letters represent statistically different means.

Furthermore, the changes in cell size and cell shape do not cause changes in the RPUF density, as shown in Figure 4. Density plays an essential role in many applications since RPUFs are widely known by their high specific mechanical properties. Moreover, the apparent density (Figure 5) is an indirect measurement of void content, and the higher the void content, the higher the gas content and the higher the insulation properties [32]. Considering that some recent studies ascribed increases in RPUF density to similar fillers being incorporated into the RPUFs [32], in this study, the inserted fillers probably did not change the RPUF density due to their low content (c.a. 2.5 wt%).

### 3.3. Compressive Properties

**Figure 6:**
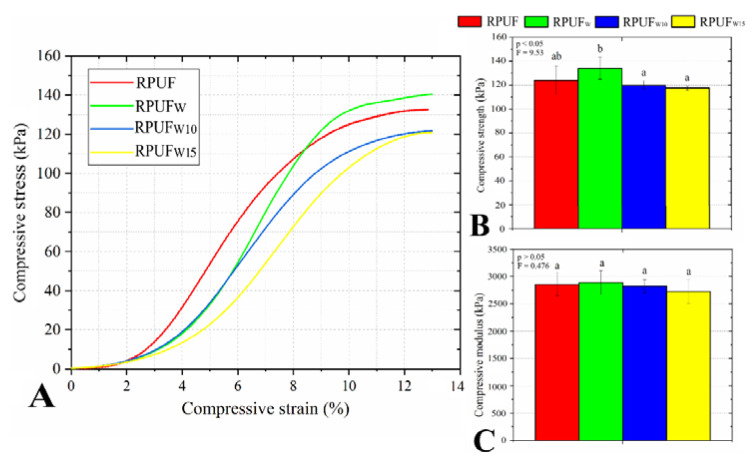
Representative stress vs. strain curves (**A**) and compressive properties (**B**, **C**) of the studied RPUFs. Different letters represent statistically different means.

Regarding the compressive stress, the use of fillers in the RPUFs did not modify the shape of the stress × strain curves obtained by compressive tests (Figure 6a), which also explains why the insertion of the fillers changed neither the compressive modulus nor the compressive strength (Figure 6b). On the other hand, the compressive strength of the RPUF reinforced by wood flour overcame those of the RPUFs incorporated with treated fillers (RPUF_W10_ and RPUF_W15_). This probably occurred due to the OH-blocking caused by the furfurylation, which probably led to a smaller amount of urethane linkages between the filler and the matrix [33]. In addition, the rounded cell shape of the RPUF filled with pristine wood flour may be a more mechanically stable configuration than the elliptical shape of the RPUFs incorporated with treated wood flour. These results are in agreement with those reported in the literature with filler percentages close to the present study. Strakowska and co-workers [34] manufactured RPUFs reinforced with a sugar beet pulp at three different concentrations (1, 2, and 5 wt.%). These authors also reported similar compressive properties in a comparison between the filled and unfilled RPUFs.

On the other hand, some studies on filled RPUFs reported losses in mechanical properties ascribed to disruptions in cell edges caused by the fillers. In this sense, Ju et al. [18] and Bradai et al. [19] reported decreases in compressive strength in RPUFs filled with steam-exploded peanut shell fibers and wood flours, respectively. These changes in mechanical properties were also attributed to the changes in density, which did not occur in the present study.

### 3.4. Thermal Stability

**Figure 7:**
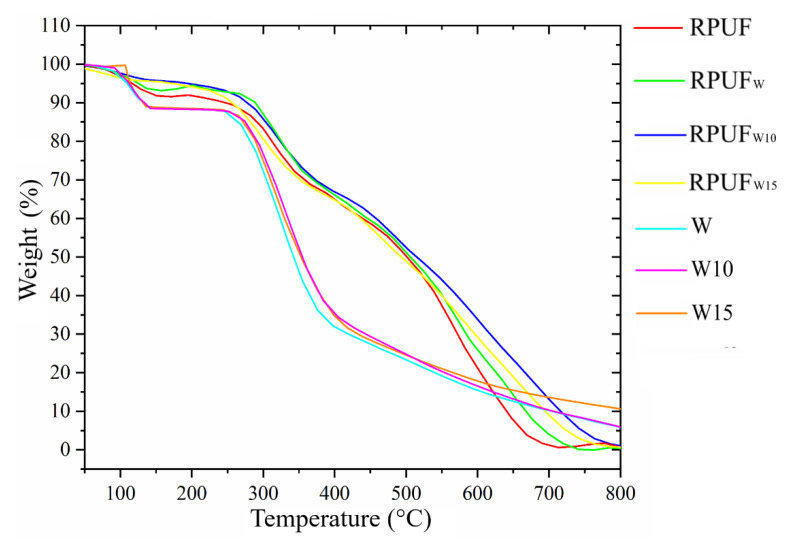
TG curves of the studied fillers and RPUFs.

The TG results are presented in Figure 7 and Table 2. The studied fillers present similar TG curves, which means that this analysis did not detect effects attributed to the wood furfurylation. In this regard, the first thermo-decomposition stage ended at 100 °C and can be attributed to a small amount of moisture retained in the wood structure. The second stage, from 100 °C to 250 °C, is attributed to the decomposition of amorphous regions from cellulose and hemicellulose [35]. In this temperature range, the thermal degradation of AF also begins with the scission of methylene bonds and the formation of certain compounds, such as 2-methyl furan and 2-furfuryl-5-methylfuran [36]. However, since the addition of furfuryl alcohol was relatively low, no significant differences were found in a comparison between the wood samples. The third TG stage, over 250 °C, can be attributed to the decomposition of crystalline cellulose and lignin units [12].

The TG results of the RPUFs confirm the FTIR results and again indicate that all the studied RPUFs have a similar chemical composition, which also means that the incorporated fillers did not insert a significant chemical modification in the RPUF cell structure. This can also be explained by a slight uneven distribution of the filler in the RPUF matrix since the sample prepared for this analysis is too small. Therefore, the different crosslinking densities indicated by the wet-chemical and FTIR results were not detected by the TG analysis. The characteristic temperatures were defined as T_2%_ (temperature attributed to 2% of weight loss), T_5%_ (temperature attributed to 5% of weight loss), and T_50%_ (temperature attributed to 50% of weight loss).

### 3.5. Water Uptake

**Figure 8:**
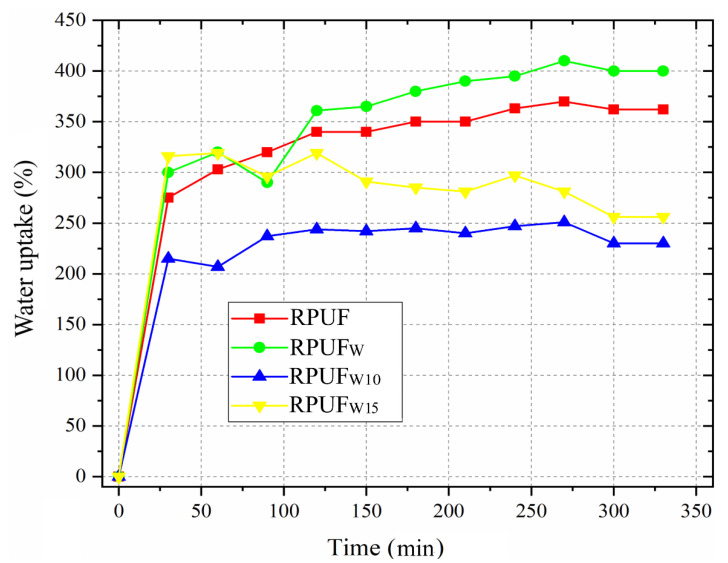
Water uptake of the studied fillers and RPUFs.

Figure 8 shows that, compared to the neat RPUF, both wood flours treated by furfurylation yield a decrease in water uptake behaviors, especially after 100 min of water exposure. According to Gu et al. [33], when chemically compatible fillers are incorporated into the RPUF system, there is a formation of new small bubbles, which may encapsulate blowing gas and impair water filling. Besides that, Członka et al. [37] stated that the morphological characteristics of the cellular structure of RPUFs are the main influential factor due to their hydrophobic/hydrophilic character. In this sense, as discussed above, the anisotropic cells imparted by the fillers may be influenced by the obtained water uptake results. In all, even a similar moisture uptake can be considered a favorable result for a filled RPUF since absorbed water may have a detrimental effect on the mechanical, thermal, and acoustic properties of the RPUF.

## 4. Conclusions

furfuryl alcohol was successfully used for treating the wood flour based on the FTIR results, yielding a decreased surface wettability. The SEM images reveal that the insertion of treated fillers yield more elliptical cells, which are different from the rounded cells of the neat RPUF. The insertion of treated fillers influences neither the apparent density nor the compressive properties, which is considered a favorable result and indicates that the fillers did not disrupt polymer cell edges. Compared to the neat RPUF, slight increases in thermal stability are found for the filled ones, especially above 500 °C. Finally, there are decreases in hygroscopic performance, which is attributed to the insertion of the treated fillers into the RPUFs, which may be of interest for several applications. These gains seem to be related to the binding of OH groups from the fillers by furfurylation. Further studies may address tensile tests and SEM images of the fractured surfaces.

## Figures and Tables

**Table 1:** Nomenclature of the studied RPUFs.

Group	Filler Weight Fraction (%)	FA Weight Fraction (%)
PU	0	0
RPUF_W_	2.5	0
RPUF_W10_	2.5	10
RPUF_W15_	2.5	15

**Table 2:** Main TG events of the studied fillers and RPUFs.

Group	T_2%_ (°C)	T_5%_ (°C)	T_50%_ (°C)	Residue at 600 °C (%)
RPUF	84.88	114.81	500.58	21.26
RPUF_W_	91.38	125.24	504.69	25.94
RPUF_W10_	89.32	187.31	514.94	33.88
RPUF_W15_	67.27	94.96	494.59	29.23
W	91.55	108.65	342.40	15.40
W_10_	97.70	112.76	355.23	16.77
W_15_	108.65	113.10	351.98	17.85
